# Viral and Host Genetic and Epigenetic Biomarkers Related to SARS-CoV-2 Cell Entry, Infection Rate, and Disease Severity

**DOI:** 10.3390/biology11020178

**Published:** 2022-01-23

**Authors:** Jernej Gaspersic, Vita Dolzan

**Affiliations:** Pharmacogenetics Laboratory, Institute of Biochemistry and Molecular Genetics, Faculty of Medicine, University of Ljubljana, Vrazov trg 2, 1000 Ljubljana, Slovenia; jernej.gaspersic@mf.uni-lj.si

**Keywords:** biomarkers, COVID-19, epigenetics, genetics, miRNA, polymorphisms, SARS-CoV-2

## Abstract

**Simple Summary:**

COVID-19 emerged as a new disease with quick transmission and a high mortality rate at the end of 2019, caused by SARS-CoV-2. Common features of the coronavirus family helped resolve structural and entry mechanism characteristics of SARS-CoV-2. Still, rapid mutagenesis leads to the fast evolution of the virus and the emergence of new strains that differ in infectivity, morbidity, and mortality. Besides differences in the viral genome, genetic variability in the host defense and immune systems may also play a role in the outcome of virus–host interactions. Furthermore, epigenetic mechanisms may also influence the outcomes, including miRNA gene silencing and DNA methylation, which may be heavily influenced by SARS-CoV-2. Molecular biomarkers are intensively investigated as potential predictive and prognostic biomarkers of the disease course and treatment response. We reviewed new data regarding the mechanisms behind fast virus mutagenesis, infectivity, and potential human genetic and epigenetic characteristics that may lead to a more severe or lethal outcome of the disease.

**Abstract:**

The rapid spread of COVID-19 outbreak lead to a global pandemic declared in March 2020. The common features of corona virus family helped to resolve structural characteristics and entry mechanism of SARS-CoV-2. However, rapid mutagenesis leads to the emergence of new strains that may have different reproduction rates or infectivity and may impact the course and severity of the disease. Host related factors may also play a role in the susceptibility for infection as well as the severity and outcomes of the COVID-19. We have performed a literature and database search to summarize potential viral and host-related genomic and epigenomic biomarkers, such as genetic variability, miRNA, and DNA methylation in the molecular pathway of SARS-CoV-2 entry into the host cell, that may be related to COVID-19 susceptibility and severity. Bioinformatics tools may help to predict the effect of mutations in the spike protein on the binding to the ACE2 receptor and the infectivity of the strain. SARS-CoV-2 may also target several transcription factors and tumour suppressor genes, thus influencing the expression of different host genes and affecting cell signalling. In addition, the virus may interfere with RNA expression in host cells by exploiting endogenous miRNA and its viral RNA. Our analysis showed that numerous human miRNA may form duplexes with different coding and non-coding regions of viral RNA. Polymorphisms in human genes responsible for viral entry and replication, as well as in molecular damage response and inflammatory pathways may also contribute to disease prognosis and outcome. Gene ontology analysis shows that proteins encoded by such polymorphic genes are highly interconnected in regulation of defense response. Thus, virus and host related genetic and epigenetic biomarkers may help to predict the course of the disease and the response to treatment.

## 1. Introduction

COVID-19 emerged as a new disease with quick transmission and a high mortality rate at the end of 2019, caused by SARS-CoV-2. In mid-2020, the pandemic was declared. Since then, more than 200,000 scientific papers have been published on COVID-19.

**Figure 1:**
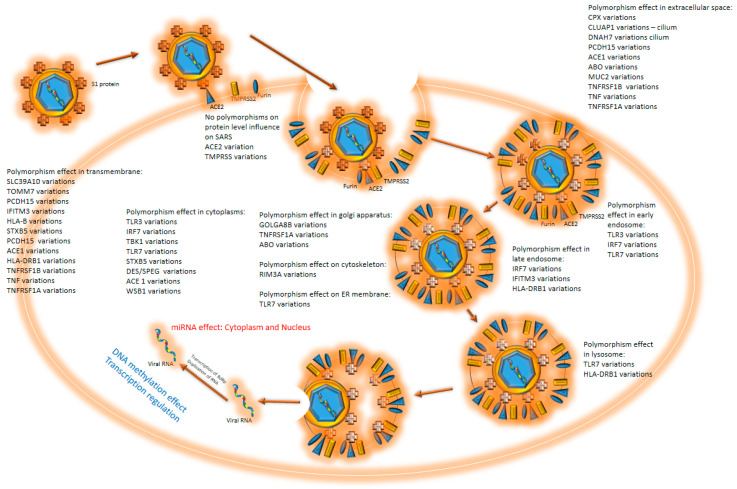
COVID 19 cell entry: virus enters the cell through interaction with ACE2 protein. Upon binding, the protease cleaves S1 protein. S1 interacts with different cell membrane proteins, which causes the membrane to envelop the virus, accelerating endocytosis. The endosome goes through all phases to the lysosome, where RNA is released from the endosome and enters the cytoplasm. At all stages, polymorphisms may influence the virus entry and duplication. When virus RNA is released into the cytoplasm, miRNA effects step in. Virus offensive mechanisms contra cell defense efforts and continue to duplicate. Human DNA methylation effects should be noticed throughout the process.

SARS-CoV-2 enters the cell through the binding of S1 proteins with the help of the *ACE2* receptor [1,2] (Figure 1). TMPRSS2 and furin are necessary for proteolytic activation of the virus SARS-CoV-2 [3], since furin and TMPRSS2 inhibitors were shown to block SARS-CoV-2. [3]. TMPRSS2 cleaves S1/S2 at the cleavage site in SARS-CoV-2, a process requiring pre-cleavage by furin [4,5]. Once inside the lysosome, the viral envelope gets degraded with lysosome protease. RNA-dependent RNA polymerase (RdRp) is first to be translated. With the help of RdRp, a negative strand of RNA is produced that serves as a template for the multiplication of viral RNA. It was suggested that SARS-CoV-2 RNAs could be reverse transcribed and integrated into the human genome [6]. The large CpG island (18 CpG) in region 151–368 could contribute to the regulation of the expression of the viral template at the appropriate time for virus reactivation (UCSC Genome Browser, CpG islands, http://genome.ucsc.edu/ (accessed 15 December 2021)).

From the cytoplasm, where proteins are translated, proteins get transported into the endoplasmic reticulum (ER), where they are post-translationally modified and transported towards the Golgi apparatus (GA) via the intermediate compartment (IC) [7]. IC virion is assembled by wrapping virion proteins around N protein, bound to viral RNA. The assembled viral particles may be exocytosed from the cell [2,8]. SARS-CoV-2 was detected in multiple organs of a COVID-19 patient who had died because of a multiorgan failure. Besides the respiratory system (e.g., lungs and trachea), it also infected the kidneys, small intestines, pancreas, blood vessels, and other tissues, such as sweat glands and vascular endothelial cells in the skin [9].

SARS-CoV-2 replicates more actively and effectively in human lung tissues than SARS-CoV; a higher viral load was found likely due to ongoing immune evasion mechanisms or defective viral clearance [10]. Mutations in the S1 protein or other regions involved in binding and entry into the human cell have been associated with the infectivity of a different strain of SARS-CoV-2.

Patients infected with SARS-CoV-2 may have no symptoms or develop a critical illness. Five different categories of severity exist according to the NIH data, as follows: (1) asymptomatic, pre-symptomatic infection—positive test and no symptoms; (2) mild illness with symptoms—fever, cough, sore throat, malaise, headache, muscle pain, nausea, vomiting, diarrhoea, and loss of taste and smell—but without shortness of breath, dyspnoea, or abnormal chest imaging; (3) moderate illness with lower respiratory disease and oxygen saturation ≥ 94%; (4) severe illness with oxygen saturation under 94%, the ratio of arterial partial pressure of oxygen to fraction of inspired oxygen < 300 mm, a respiratory rate > 30 breaths/min, or lung infiltrates > 50%; (5) critical illness with respiratory failure, septic shock, and/or multiple organ dysfunction [11].

COVID-19 symptoms are fever, a dry cough, dyspnoea, myalgia, and pneumonia. Some patients also reported sore throat, rhinorrhoea, headache, and hyposmia [12]. COVID-19 patients that needed hospitalization or intensive care unit (ICU) presented with pneumonia with fever, lymphopenia, highly elevated pro-inflammatory cytokines, C-reactive protein (CRP), serum ferritin, and D-dimers [13]. Plasma levels of IL2, IL7, IL10, GSCF, IP10, MCP1, MIP1A, and TNFα were higher in ICU patients compared with non-ICU patients [14].

Genetic predisposition for viral infection or disease progression has been proposed/suggested. Biomarkers can alarm the medical doctors to susceptibility or resistance of the patient towards SARS-CoV-2. Human biomarkers can be used to detect and predict the severity or life-threatening condition of COVID-19 disease. Disease severity can be foreseen prior to infection with COVID-19.

In this review, we have performed a literature and database search to summarize potential viral and host-related genomic and epigenomic biomarkers, such as genetic variability, miRNA, and DNA methylation in the molecular pathway of SARS-CoV-2 entry into the host cell, that may be related to COVID-19 susceptibility and severity. 

## 2. Virus Mutations

### 2.1. Virus Strains

Viral mutations and recombination gave birth to new strains that may have different reproduction rates or infectivity and may impact the course and severity of the disease.

The COVID-19 virus strains were named after Greek alphabetical letters, and the designation is based on the positions and number of mutations. There are some disagreements regarding mutations belonging to specific strain groups, probably because different mutations evolved and spread further on different continents and states. Mutations labeled with * are present in some strains [15]. The Alpha variant has mutations of sites E484K*, D614G, delH69V70, and N501Y. Next, the Beta strain, characterized by E484K, D614G, A701V, N501Y, L242_244L, and K417N mutations, and the Gamma strain, with mutations of sites E484K, D614G, K417T, N501Y, and T20, evolved, and both of them outcompeted the wild-type strain [16]. The Delta variant, with mutations of sites D614G, L452R, P681R, and T478K emerged in April 2021. In July 2021, the Delta variant outcompeted all the other strains [16]. Iota has mutations of sites A701V*, E484K*, L452R*, and D614G; Epsilon has mutations of sites L452R and D614G; Eta has mutations of sites E484K, D614G, and delH69V70; Kappa has mutations of sites E484Q, D614G, L452R, and P681R. 

Another classification was made with slightly different mutations: Beta (B.1.351) has mutations of sites N501Y, E484K, K417N; Alpha (B.1.1.7) has mutations of sites N501Y, E484K, and HV69/70del; Gamma (P.1) has mutations of sites N501Y, E484K, K417T, and V1176F; Delta (B.1.617.2) has mutations of sites N501, E484, and (L452R, P681R, T478K) [17]. The third classification defines that the Delta strain holds L452R and T478K mutations, the Epsilon (B.1.427 and B.1.429) strain holds L452R mutations, and the Kappa (B.1.617.1) strain holds L452R and E484Q mutations [18].

A new Omicron variant (B.1.1.529) emerged at the end of November 2021 [19]. It is classified as a variant of concern (next to Beta, Gamma, and Delta) and holds 33 spike protein mutations, many of which were found in the Alpha and Delta strains [19].

### 2.2. Virus Mutations Position and Their Influence on SARS 2 Disease Development

SARS-CoV, which has a similar structure and RNA sequence to SARS-CoV2, had an estimated mutation rate of approximately 0.80–2.38 × 10^–3^ nucleotide substitutions per site per year, and the non-synonymous and synonymous substitution of approximately 1.16–3.30 × 10^–3^ and 1.67–4.67 × 10^–3^ per site per year, respectively, which is similar to other RNA viruses [20]. The large CoV RNA genome allows modification by introducing ‘’non-lethal’’ mutations and recombination, leading to increased probability for intraspecies variability, interspecies “host jump”, and novel CoVs to emerge [20]. SARS-CoV-2 has a higher fidelity in its transcription and replication process than other single-stranded RNA viruses because it has a proofreading mechanism, regulated by NSP14. However, despite this mechanism, the mutation rate is very high [21].

The mutation rate of SARS-CoV-2 is so high that it may impact diagnostic test accuracy [21]. In summary, the target spike and other SARS-CoV-2 proteins have numerous mutations. In total, 13,402 single mutations were found among 31,421 virus isolates, many of them located in coding regions currently used for COVID-19 diagnostic tests [21]. 

**Figure 2:**
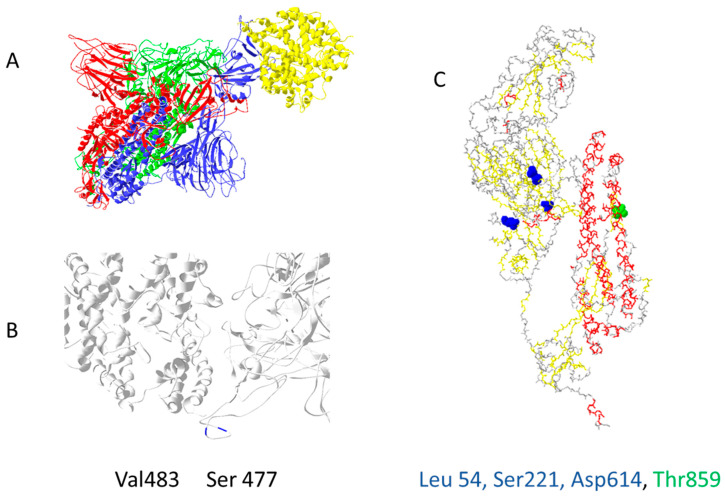
The most common mutation sites on activated spike protein: (**A**) trimer of activated spike protein (green, blue, red) interacts with ACE2 receptor (yellow); (**B**) two common mutation sites, V483 and S477 (dark blue), are close to the ACE2 interaction site; (**C**) three mutations should impact the 3D structure of spike proteins L54, S221, D614; T859 is located near spike trimer interaction site, PDB file—7DF4 [24].

Out of 400 distinct mutation sites of spike protein, 10 mutation sites are most commonly mutated: D614(7859), L5(109), L54(105), P1263(61), P681(51), S477(57), T859(30), S221(28), V483(28), and A845(24) [22]. In Figure 2, mutations positions are presented on the 3D structure of activated mono-trimer spike protein (Figure 2A). Spike protein extracellular domain is divided into the S1 and S2 domains, on which RBD resides, and HR is divided into two domains, which connect S domains with the transmembrane region [23]. Mutations 483 and 477 are located on the loop close to the ACE2 binding site (Figure 2B). The rest are spread throughout the 3D structure of the spike protein, as shown in Figure 2C. Figures were prepared as described in the Supplementary Materials (Supplementary File S1).

**Figure 3:**
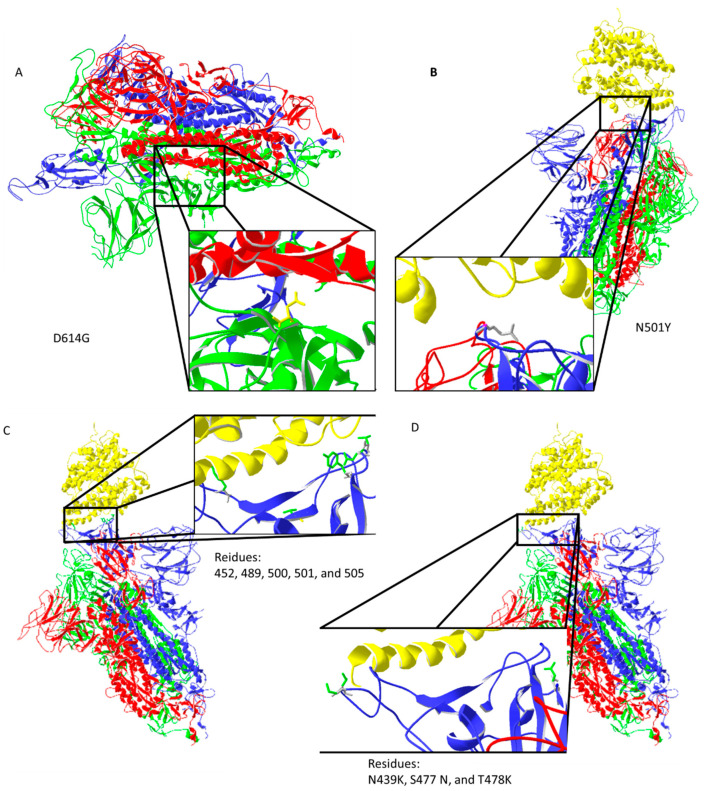
Important mutation sites of activated spike protein that increase or could increase the infectivity of the virus: (**A**) D614G mutation may increase interaction between trimer of spike protein (the negatively charged amino acid is replaced by neutral glycine that interacts with the opposite chain, where aliphatic amino acids are present); (**B**) N501Y mutation should reduce repulsive forces with ACE2; (**C**) mutations in the ACE2 interaction region could potentially increase the infectiveness of the virus; (**D**) predicted amino acid replacements that should increase binding with ACE2 are localized on the ACE2 binding site. Protein data bank (PDB) file: 7DF4 [24].

Spike protein D614G mutation increases virus entry (Figure 3A) by enhancing the binding properties to the ACE2 protein [25]. Increased transmission is predicted for N501Y mutation [26] (Figure 3B). Residues 452, 489, 500, 501, and 505 on the RBM of spike protein (Figure 3C) have high chances of mutating into more infective strains [27]. Suleman et al. computed three mutations, N439K, S477 N, and T478K, shown in Figure 3D, to increase binding with ACE2 [28]. These mutations are localized on, or close to, the ACE2 binding site, except for D614G, which indirectly enhances ACE2 interaction through spike trimer binding enhancement (Figure 3). Bioinformatic predictions data match strain infectivity data. Mutations in strains overlap with the mutations predicted by bioinformatics tools [26,27,28]. 

Information regarding 3D structure and mutagenesis is getting more accurate, which helps in drug development. Fast mutagenesis helps the virus evolve much faster than the human defense system can adapt to.

## 3. Virus–Host Interactions Affecting Viral Replication and Transcription

Viral infection triggers several mechanisms that are both virus- and host-dependent. On the one hand, viral replication affects transcription factors that promote viral replication; on the other hand, the host defense mechanism tries to activate factors to stop the virus from replicating itself.

SARS-CoV-2 targets transcription factors E2F1, SP1, EIF4A1, and TBP, and tumour suppressor genes, including PTEN, AKT1, and RB1, to influence the expression of different host genes (MAPK1, MAPK3, MAPK4, MAPK6, MAPK7, PIK3CA, and CAMK). For regulation, SARS-CoV-2 uses miRNAs [29]. Virus influence on RELA (NF-κB activation), E2F1, STAT3, TP53, NFKB1, GATA3, and CREB1 may impact the regulation of disease progression [30].

One CpG island with 18 CpG sites was detected at the start of viral RNA (UCSC Genome Browser). This site could impact the replication process and translation of proteins coded at the start of the RNA. If COVID-19 integrates into human DNA, this CpG island could impact when and how virus reactivation would start.

## 4. Human Polymorphism May Influence SARS-CoV-2 Viral Entry and Replication

Natural genome diversity contributes to the survival of the species. Polymorphisms in human genes responsible for viral entry and later replication may contribute to disease prognosis and outcome (Figure 1). These polymorphic genes, which may influence the course of COVID-19 disease, have different roles in viral entry and replication (Table 1).

The most investigated genes are the ones that directly interact with spike protein. Single nucleotide polymorphisms (SNPs) in *ACE2* [30,31,32,33,34,35] and *TMPRSS2* may contribute to selective binding of SARS-CoV-2 [36,37,38]. Interestingly, only the intron variant of ACE2 and two same sense variants of TMPRSS2 should influence SARS-CoV-2 entry (Table 1). Polymorphisms in the *ADAM17* gene were suggested to have a role in the outcome of the disease [37]. It was proposed that the higher frequency of *ACE* D allele contributed to higher numbers of infected patients/million and mortality rate in the Asian population [66].

Vitamin D deficiency was also associated with a more severe course of COVID-19. Polymorphisms in genes that may lead to this condition, such as vitamin D transporter (*GE*), receptor (*VDR*), and NAD synthase gene (*NADSYN1*), were associated with the critical condition [65].

It was discovered that blood type might also contribute to susceptibility to the COVID-19 disease. HLA types also contribute to susceptibility and severity. Increased susceptibility to SARS-CoV-2 was discovered in ABO A- type patients (*ABO* (A, B, and O)), e4e4 genotype (*APOE* (e3 and e4)), *HLA B*, *DRB1*, *DQB1*, and *DRB1* alleles, 3p21.31 region minor allele and novel missense variant in *GOLGA8B* rs200975425 and *RIMBP3* rs200584390 [48].

Polymorphisms in Toll-like receptors *TLR3* [48,52], *TLR4* [55], and *TLR7* [41] influence COVID-19 susceptibility and severity as these receptors play a fundamental role in innate immunity through recognition of different pathogen molecules. TLR3 recognizes double-stranded RNA in endosomes [53], TLR4 is activated by lipopolysaccharide and some viral proteins [67], while TLR7 binds to single-stranded RNA [53]. Specific polymorphisms in these receptors attenuate innate immunity response.

Polymorphisms in genes that are directly or indirectly involved in the immune defense system contribute in different ways to infectivity severity and mortality of the SARS-CoV-2 disease (Table 1).

Increased susceptibility to SARS-CoV-2 was associated with carriers of several alleles in *HLA-A*, *HLA-DRB1*, minor allele carriers *ACE2* rs61735794, and rs61735792 [48].

Several variations were associated with increased severity of SARS-CoV-2 disease—*IFITM3* (rs12252), *ACE1* (rs4646994), and *TMEM189–UBE2V1* (rs6020298)—while multiple alleles of *HLA-A, HLA-B*, *HLA-C,* and *HLA-DRB1* were associated with decreased severity [48].

Increased mortality was reported in carriers of variants in some loci—*STXBP5/STXBP5-AS1*, *CPQ*, *CLUAP1*, *WSB1*, *DNAH7/SLC39A10*, *DES/SPEG*, *TOMM7, PCDH15*—and in 10 variants in *TLR3* and *IRF7* [48].

Several additional polymorphisms were associated with COVID-19: *PCDH15* [48,53], *ABO* [48,56,57,58], *APOE* [59], *RIMBP3* [47,48], *GOLGA8B* [47,48], *C3* [60,61], *CCR5* [62], *IL37* [63], *IFNAR2* [31], *DPP9* [31], *IFNL4* [64] *TNFRSF13B* [39,40], *TNF* [39,68], *TNFRSF1A* [39], *TBK1* [39], *IFITM3* [42,43], *ACE* [44,45,46], *IRF7* [48,52], *TMPRSS2* [36,37,38], *HLA* [48,49,50,51], and *AGT* [46] (Table 1). 

Several polymorphic genes were investigated for differences in susceptibility and protection against SARS-CoV-1, which virus has similar structure, RNA sequence, and receptor binding properties to SARS-CoV-2 [69]. Important human polymorphisms were also reported in *HLA*, *ACE1*, *OAS-1*, *MxA*, *PKR*, *MBL*, *E-CR1*, *FcγRIIA*, *MBL2*, *L-SIGN (CLEC4M)*, *IFNG*, *CD14*, *ICAM3*, *RANTES*, *IL-12 RB1*, *TNFA*, *CXCL10/IP-10*, *CD209 (DC-SIGN)*, *AHSG*, *CYP4F3*, and *CCL2* [69].

Gene ontology analysis (WEBGESTALT) (Supplementary Materials (Supplementary File S1)) shows that proteins encoded by genes stated in Table 1 are highly interconnected in regulation of defense response (*p* = 4.0406e-10, enrichment ratio = 9.1490) (mostly through regulation of interferon alpha and beta (*p* = 5.1619 × 10^−10^, enrichment ratio = 277.89)). Mostly, they are localized on the cellular membrane (*p* = 0.0000054530, enrichment ration = 4.3801) or endosomal membranes (*p* = 0.00010358, enrichment ratio = 7.6820), where they combat against virus entry with the help of signal receptor activity (*p* = 0.00068370, enrichment analysis = 3.4852) and exopeptidase activity (*p* = 0.0010791, enrichment ratio = 14.740). Data were accessed as described in Supplementary Materials (Supplementary File S1). Genetic polymorphisms may change cell defense parameters and contribute to different susceptibility and later severity of disease.

### Engineered ACE2 Mutations May Predict Virus–Host Interactions

**Figure 4:**
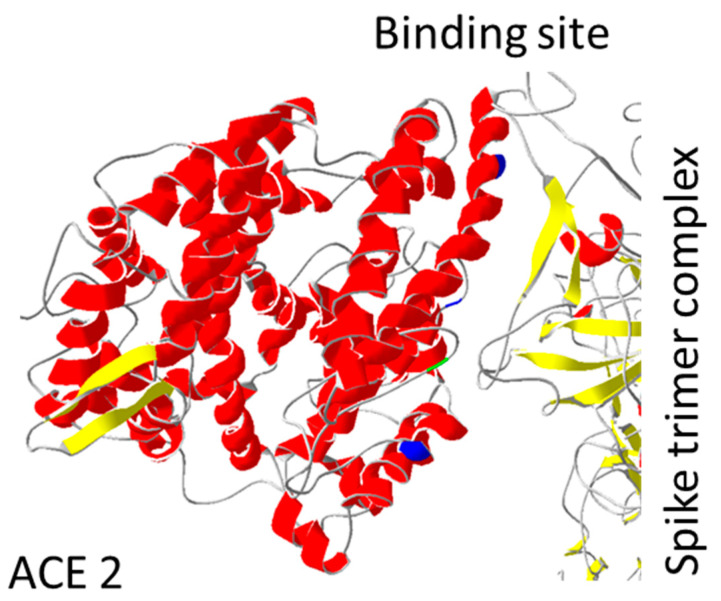
ACE2 mutations position: Mutations important for interaction with spike protein are shown. Mutation D355A is coloured green, while the trio of Y27, L330, and L386 are coloured dark blue. All mutations are localized close to spike binding site. PDB file: 7DF4 [24].

Due to the main interaction with spike protein, ACE2 protein was thoroughly investigated for increased/decreased interaction with SARS-CoV-2 spike protein. They predicted an important role of mutations in regions: 19–42; 69–92; 324–330 (Table 2). Human ACE2 receptor with Y27, L330, and L386 triple mutation showed the highest increase in interaction with RBD of SARS-CoV-2 spike protein (Uniprot database section: “Mutagenesis”). The opposite effect was observed for ACE2 D355N mutation both in vitro and in vivo [70]. ACE2 D355A mutation has a similar effect when interacting with the spike protein of SARS-CoV-1 (Uniprot database section: “Mutagenesis”). These mutations are set in or close to the spike binding site (Figure 4). 

The diversity of polymorphisms in genes with different functions indicates that the virus replication is affected in all stages, from entry, to transcription, to exocytosis (Figure 1). Knowledge of a patient’s genetic background may support informed choice of treatment.

## 5. Changes in mRNA Expression—miRNA ‘’Silencing’’ Interference

siRNA are small RNAs that interfere with RNA translation as they bind to mRNA. miRNA have a similar role and regulate gene expression in the cytoplasm. They are involved in transcriptional gene regulation and alternative splicing [71]. miRNA binding slows down replication of viral RNA and slows translation of viral proteins. Genetic variations may change either miRNA or target site sequence and thus change the expression pattern of several genes. 

The virus may interfere with RNA expression in host cells by exploiting endogenous miRNA and its viral RNA.

The SARS-CoV-2 genome targets a large number of different miRNA, as follows: transcripts of interleukins IL2, IL5, IL7, IL8, IL10, IL13, IL15, IL16, IL17, IL21, IL22, IL24, IL25, and IL33, histone demethylase genes JARID1A, JARID1C, and JARID2, and histone deacetylase genes, including HDAC1, HDAC2, and HDAC3 [29].

Four key miRNAs (hsa-miR-342-5p, hsa-miR-432-5p, hsa-miR-98-5p, and hsa-miR-17-5p) are believed to be involved in antiviral SARS-CoV-2 gene silencing (ORF1ab) [72]. For three miRNA (hsa-miR-17-5p, hsa-miR-20b-5p, and hsa-miR-323a-5p), there is experimental evidence of having antiviral roles during infections against SARS-CoV-1 and SARS-CoV-2 (Table 3) [73].

7c-5p, miR-27b-3p, miR-98-5p and miR-125a-5p target COVID-19 genome; let-7b-5p, miR-155-5p, miR-186-5p, miR-16-5p, miR-27b-3p, miR-29a-3p, and miR-30a-5p are associated with development of COVID-19 symptoms (Table 3) [30].

Importantly, seven key microRNAs were identified (miRNAs 8066, 5197, 3611, 3934-3p, 1307-3p, 3691-3p, and 1468-5p) that may be linked to human response and virus pathogenicity (Table 3) [40].

A total of 22 potential SARS-CoV-2 miRNAs, found in 5 different genomes, targeted 12 human miRNAs. Binding sites for human miRNAs hsa-mir-1267, hsa-mir-1-3p, and hsa-mir-5683 were predicted in all 5 viral SARS-CoV-2 strain genomes (Table 3) [74].

Many studies have shown that miRNAs were significantly dysregulated in COVID-19 [75]. The most important miRNAs that can be used as COVID-19 biomarkers are miR-21-5p, miR-144, and miR-155 [75]. miRNA-146a can be used as a biomarker for the severity of COVID-19 [75].

Our search of miRBASE (mirbase.org) identified a new set of 24 miRNAs that may bind to viral RNA: hsa-miR-1468-5p, hsa-miR-378c, hsa-miR-3611, hsa-miR-3914, hsa-miR-3120-5p, hsa-miR-10397-5p, hsa-miR-515-5p, hsa-miR-584-3p, hsa-miR-4502, hsa-miR-190b-5p, hsa-miR-3691-3p, hsa-miR-597-3p, hsa-miR-1287-5p, hsa-miR-148b-3p, hsa-miR-3672, hsa-miR-5197-3p, hsa-miR-3934-3p, hsa-miR-129-2-3p, hsa-miR-3085-3p, hsa-miR-8076, hsa-miR-8066, hsa-miR-1307-3p, hsa-miR-3613-5p, and hsa-miR-1270 (Table 4). miR-3914, 515-5p, 5197-3p, 4502, 584-3p, 3120-5p, 8066, 5197-3p, and 1287-5p have more than one binding site. miR-3914, 515-5p, 3934-3p, 8076, 4502, and 584-3p have a higher chance of duplexing (https://sfold.wadsworth.org/cgi-bin/index.pl (accessed 15 December 2021)). miRNA regulate numerous human genes (www.TargetScan.org (accessed 15 December 2021)), only a selected few are stated in Table 4. Data were collected as described in Supplementary Materials (Supplementary File S1). During infection, host miRNA may be silenced by binding to viral RNA, disrupting normal cell function through up-regulation of gene expression.

**Figure 5:**
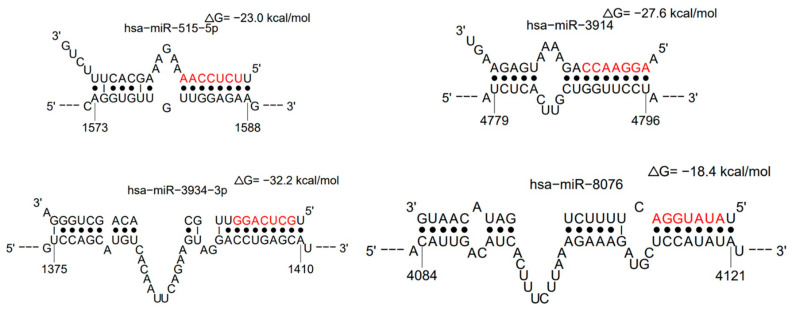
Examples of alignments of miRNAs duplexes with viral RNA—hsa-miR-515-5p, hsa-miR-3914, hsa-miR-3934-3p, and hsa-miR-8076—have the highest probability of duplex formation and high bond enthalpy (sFOLD- presentation of duplexes). High bond energy needs to be overcome to allow viral RNA duplication and translation of proteins.

Analysis of miRNA data with sFOLD shows that several RNAs may have more than one binding site on viral RNA (Table 4). The duplex formation was evaluated in probability and bond energy enthalpy (Figure 5).

The virus attacks the cell through miRNA and subdues it for faster viral replication.

Published data show that virus RNA highly interacts with human miRNAs to change the expression of important defense molecules [29,72,73]. Our analysis showed that numerous human miRNA may form duplexes with different coding and non-coding regions of viral RNA. These interactions could disturb important virus proteins’ replication and/or translation. RNA polymerase and ribosomes require additional energy to remove duplexes during RNA duplication or protein synthesis.

### Changes in DNA Methylation Profile

Coronaviruses can delay pathogen recognition and block interferon-stimulated genes [76]. Several known viral proteins associated with viral pathogenesis are controlled epigenetically [76]. Viruses such as Epstein–Barr virus and SARS-CoV-2 can demethylate the syncytin 1 and 2 genes, resulting in the augmentation of gene transcription [76]. Hypo-methylation of ACE2 coupled with demethylation of interferon- and cytokine-regulated genes and enhanced NF-κB axis have been shown to contribute to SARS-CoV-2 disease severity [76]. Critically ill COVID-19 patients had hypermethylation of IFN related genes and hypomethylation of inflammatory genes [76].

An epigenome-wide COVID-19 study reported 51 CpG sites with different methylation profiles between moderate and severe cases [77]. After thorough analysis, 44 CpGs were marked as important, as follows: 15 CpG sites were located in human genomic regions with no currently described gene sequence; 6 CpG sites were associated with non-coding RNA; 23 CpG sites were located within 20 known coding genes. In 17 out of 20 coding genes (85%), the presence of hyper-methylation was significantly associated with transcript down-regulation—7 out of 20 were effectors of interferon signalling, as follows: *AIM2*, *HLA-C*, *IFI44L*, *CXCR2*, *KIFAP3*, *SGMS1,* and *VIM* [77]. Two CpG methylation sites were found in *PM20D1, AIM2,* and *HLA-C* [77].

Methylation of DNA may influence virus replication, and vice versa; the infection may start to change the methylation profile of patients. Better knowledge of epigenetic markers could perhaps help predict the course of the disease.

## 6. Extracellular Vesicles as Biomarkers

Extracellular vesicles (EVs), such as exosomes and microvesicles, could be used as biomarkers if sufficiently up- or down-regulated in COVID-19 patients. Several lipid molecules, GM3, and sphingomyelins were enriched in exosomes, while diacylglycerol levels were decreased [78].

Several RNA species were detected in large quantity in exosomes (exRNAs), such as snRNAs SNORD33, RNU2-29P; transcripts AL732437.2 and AL365184.1; non-coding RNAs CDKN2B-AS1, and miRNAs miR-122-5p [79], hsa-miR-146a and hsa-miR126-3p [80].

Proteins such as fibrinogen, fibronectin, complement C1r subcomponent, and serum amyloid P-component were downregulated in EVs [81].

EVs have numerous receptors on the surface: CD9, CD63, CD81, ESCRT, TSG101, Alix, Hsp60, Hsp70, Hsp90, and RAB27a/b—among them, ACE2. ACE2 on EVs may divert the virus from entering the cell, modulating or mediating SARS-CoV-2 infection [79].

EVs research is new, and it is expected that several new diagnostic methods will be developed, especially in virology and immunology.

## 7. Conclusions

This review described COVID-19 cell viral and host parameters that may influence SARS-CoV-2 entry and infectivity, along with factors that influence the susceptibility and severity of the COVID-19 disease. Virus mutations, strains, changes in transcriptome, miRNA ‘’silencing’’ interference, methylation profiles (epigenetics), and individual polymorphisms were reviewed.

COVID-19 appeared at the end of 2019, and several new strains have already been discovered. The new strain can overcome other strains in a few months. New mutations in the RBD domain can develop the virus into more infectious strains that cause diseases with more severe symptoms during infection. Sequencing analysis shows that beneficial mutations remain in the sequence of virus via outcompeting the wild-type

Virus replication may also be influenced by host genetic variability during all phases from the entry and transcription to the final stage. Several human polymorphisms, miRNAs, and methylation profiles led to different susceptibility, severity, and even mortality of the disease. In the future, complete genome sequence information may support a professional, personalized medicine approach to treat and diagnose SARS-CoV-2.

Knowledge of genetic and epigenetic biomarkers may help predict the course of the disease and the response to treatment. Research of extracellular vesicles is also on the rise, so new COVID-19 biomarkers are anticipated due to differences in vesicle composition.

## Figures and Tables

**Table 1:** Updated list of polymorphisms that influence on SARS2 susceptibility, severity, and mortality: information regarding gene name, variation, effect, reference, and function (NCBI subsection gene) are stated in the table. Stated genes: OAS [31], ACE2 [32,33,34,35], TMPRSS2 [36,37,38], TNFRSF1B (ClinVar database [39]), TNF [39], TNFRSF1A (ClinVar database [39]), TBK1 (ClinVar database [39]), TNFRSF13B (ClinVar database [39,40]), TLR7 [41], IFITM3 [42,43], ACE [44,45,46], TMEM189–UBE2V1 [47], HLA [48,49,50,51], TLR3 [48,52,53], IRF7 [48,52], STXBP5/STXBP5-AS1 [48,54], CPQ [48,54], CLUAP1 [48,54], WSB1 [48,54], DNAH7/SLC39A10 [48,54], DES/SPEG [48,54], TOMM7 [48,54], PCDH15 [48,54], TLR4 [55], ABO [48,56,57,58], APOE [59], RIMBP3 [47,48], GOLGA8B [47,48], C3 [60,61], CCR5 [62], IL37 [63], IFNAR2 [31], DPP9 [31], IFNL4 [64] GE [65], NADSYN1 [65], VDR [65] AGT [46].

Gene	rs Code	Protein Mutation	DNA Mutation	Function SARS-CoV-2	Polyphen-2	Protein Function	Reference
*ACE2*	rs2285666	Intron variant	g.14934G > T	Increased severity of SARS-CoV-2 (GG genotypes or G-allele carriers), 3 times higher risk, fatality risk		Angiotensin-converting enzyme and functional receptor of COVID-19.	[32,33,34,35]
	rs2074192	Intron variant	g.42492G > A	Increased severity of SARS-CoV-2			
		V749V	c.2247G > A	Possible protective role against SARS-CoV-2 (found in healthy controls)			
*TMPRSS2*	rs12329760	V197M	c.589G > A	Association with COVID-19 case fatality rate	0.999- probably damaging	Serine protease also facilitates entry of the COVID-19 virus by proteolytic cleaving and activation.	[36,37,38]
	rs61735792	P100P	c.300C > T	Significant association with SARS-CoV-2 infection			
	rs61735794	G422G	c.1266G > A	Significant association with SARS-CoV-2 infection			
*TNFRSF1B*		M196R	c.587T > G	Susceptibility to severe coronavirus disease (COVID-19)	0-benign	A member of the TNF receptor superfamily; its ligand, cytokine TNF, is involved in regulating a broad spectrum of processes. Mutations in this gene affect susceptibility to several diseases.	[39]
		3’ prime UTR	c.*215C > T	Susceptibility to severe coronavirus disease (COVID-19)			
*TNF*	rs1800629	Upstream variant	c.-488G > A TNFα-311A > G	Susceptibility to severe coronavirus disease (COVID-19)		Cytokine is involved in the regulation of a broad spectrum of processes. Mutations in this gene affect susceptibility to several diseases.	[39]
			g.31575324G > A	Susceptibility to severe coronavirus disease (COVID-19)			
	rs909253	Upstream variant	TNFβ-252A > G	Is more frequent in COVID-19 patients, conferrers protection against COVID-19 susceptibility			
*TNFRSF1A*		Intron variant	c.625 + 10A > G	Susceptibility to severe coronavirus disease (COVID-19)		A member of the TNF receptor superfamily of proteins. Mutations in this gene may be associated with a few diseases.	[39]
		P12P	c.36A > G	Susceptibility to severe coronavirus disease (COVID-19),			
*TBK1*		splice donor variant	c.1760 + 4 1760 + 7del	Severe SARS-CoV-2 infection, susceptibility to		Codes protein, an important kinase for antiviral innate immune response, is similar to IKB.	[39]
*TNFRSF13B*		C104R	c.310T > C	Severe SARS-CoV-2 infection, susceptibility to,	1.000-probably damaging	A member of the TNF receptor superfamily, it plays an important role in humoral immunity.	[39,40]
*TLR7*		Q710fs	c.21292132del	Preliminary severe COVID-19-related		Receptor senses ss RNA oligonucleotides with guanosine- and uridine-rich sequences.	[41]
		V795F	c.2383G > T	Immunodeficiency 74, COVID-19-related, X-linked	0.997-probably damaging		
*IFITM3*	rs12252	S14S	c.42T > C	Increased severity of SARS-CoV-2 disease in the studied Chinese cohort, the C allele of IFITM3 rs12252 polymorphism was negatively correlated with the SARS-CoV-2 mortality rate per million		Interferon-induced membrane protein helps build up immunity against several viruses.	[42,43]
	rs34481144	5 Prime UTR Variant	c.-23C > T	Were significantly more frequent in COVID-19 hospitalized patients			
*ACE1*	rs4646994	Intron variant	c.2306-109_2306-108ins	Might Increase susceptibility to SARS-CoV-2 disease ACE I/D polymorphism (Czech first-wave SARS-CoV-2-positive survivors); decreased risk of infection		An enzyme that catalyzes the conversion of angiotensin I into active angiotensin II regulates homologous ACE2, which may influence the progression of SARS-CoV-2 disease.	[44,45,46]
	rs1799752	Intron variant	g.16471_16472ins	Might facilitate human cell entry of SARS-CoV-2 due to increased level of ACE2			
*TMEM189–UBE2V1*	rs6020298	Intron variant	g.48769055G > A	Increased severity of SARS-CoV-2 disease		The function of this read-through mRNA and its protein is unknown.	[47]
*HLA*		Several variants	Several variants	HLA-B*46:01 increased severity of SARS-CoV-2; HLA DRB1*15:01 and DQB1*06:02 increased severity of SARS-CoV-2 disease; HLA-A 02-01 increased risk for COVID-19; HLA-C*04:01 associated with infection; HLA-A30:02, HLA-B 14:02; HLA-C 08:02 significant association with severe and extremely severe COVID-19		HLA molecules play a central role in the immune system by presenting antigenic peptides on the cell surface—an important role in the occurrence and outcome of SARS-CoV-2.	[48,49,50,51]
*TLR3*		p.Ser339fs		Increased life-threatening complications in patients with SARS-CoV-2 TLR3		Recognize pathogen-associated molecular patterns (PAMPs), specifically dsRNA, to combat multiple virus infections.	[48,52,53]
	rs121434431	p.Pro554Ser	c.1660C > T	Increased life-threatening complications in patients with SARS-CoV-2 TLR3	1.000-probably damaging		
		p.Trp769*		Increased life-threatening complications in patients with SARS-CoV-2 TLR3 deficiency			
		p.Met870Val		Increased life-threatening complications in patients with SARS-CoV-2 TLR3 deficiency	0.995-probably damaging		
*IRF7*		pArg7fs;	g.615095A > C	Increased life-threatening complications in patients with SARS-CoV-2 IRF7 deficiency		Plays a role in the transcriptional activation and innate immune response against DNA and RNA viruses.	[48,52]
		p.Pro364fs		Increased life-threatening complications in patients with SARS-CoV-2 IRF7 deficiency			
		p.Gln185*		Increased life-threatening complications in patients with SARS-CoV-2 IRF7 deficiency			
		p.Pro246fs		Increased life-threatening complications in patients with SARS-CoV-2 IRF7 deficiency			
*STXBP5/STXBP5-AS1*	rs116898161	Intron variant	g.147538692A > G	Increased mortality in SARS-CoV-2 disease		Protein may be involved in neurotransmitter release by stimulating SNARE complex formation.	[48,54]
	rs117928001	Intron variant	g.147514999C > A	Increased mortality in SARS-CoV-2 disease			
*CPQ*	rs1431889	Intron variant	g.98141643G > C	Increased mortality in SARS-CoV-2 disease		The enzyme catalyzes the stepwise oxidative decarboxylation of coproporphyrinogen III.	[48,54]
	rs7817272	Intron variant	g.98140470T > A	Increased mortality in SARS-CoV-2 disease			
*CLUAP1*	rs2301762	Upstream variant	g.3550977C > G	Increased mortality in SARS-CoV-2 disease		This gene contains a single coiled-coil region.	[48,54]
*WSB1*	rs60811869		g.25590833T > C	Increased mortality in SARS-CoV-2 disease		This protein has several WD-repeats and a SOCS box in the C-terminus.	[48,54]
*DNAH7/SLC39A10*	rs183712207	Intron Variant	g.196611282G > A	Increased mortality in SARS-CoV-2 disease		DNAH7 is a part of the inner dynein arm of the ciliary axonemes. SLC39A10 shows the structural characteristics of zinc transporters.	[48,54]
*DES/SPEG*	rs71040457	Upstream, downstream variant	g.220294782_220294783insG	Increased mortality in SARS-CoV-2 disease		LCR may control the preferentially muscle-expressed SPEG gene expression downstream of the DES gene.	[48,54]
*TOMM7*	rs55986907	Intron variant	g.22817292C > A	Increased mortality in SARS-CoV-2 disease		This gene encodes a subunit of the translocase of the outer mitochondrial membrane.	[48,54]
*PCDH15*	rs9804218	Intron Variant	g.56495374G > A	Increased mortality in SARS-CoV-2 disease		Plays an essential role in maintenance of normal retinal and cochlear function.	[48,54]
*OAS3*	rs10735079	Intron variant	c.460 + 351G > A	Involved in the critical illness of SARS-CoV-2 disease		This enzyme helps inhibit cellular protein synthesis and helps in viral infection resistance.	[31]
*ABO (A*, *B*, *and O)*	Set of blood type genotypes: rs1556058284; B type: rs8176743; rs8176746; rs8176747	ABO c.260insG (p.Val87_Thr88fs*); set of variants that contribute to blood type: p.Gly235Arg, p.Leu266Met, p.Gly268Val	Blood type influencing variants: del261- O type- frame shift variant; two amino acid substitutions- B type: c.703G > C, c.796C > A, c.803G > T	Increased susceptibility for SARS-CoV-2 development A type (Fan 2020, Zhao 2020), Decreased susceptibility for SARS-CoV-2 development O-type (Zhao 2020); A > O > B > AB susceptibility (Zhang 2021)		Variations in the ABO gene are the basis of the ABO blood group. Gene has a role in susceptibility and severity of coronavirus disease 2019.	[48,56,57,58]
*APOE*	rs429358 allele is (T) + rs7412(C) allele	APOE-ε4 (arg112, arg158)	c.466T > C + c.604C > T	Increased susceptibility for SARS-CoV-2 development, a 4-fold increase in mortality		It is essential for the normal catabolism of triglyceride-rich lipoproteins.	[59]
*RIMBP3*		E1263A	c.3788A > C	Increased susceptibility for SARS-CoV-2 development	0.884-possibly damaging	It may be a component of the manchette.	[47,48]
*GOLGA8B*	rs200975425	R513P	c.1538G > C	Increased susceptibility for SARS-CoV-2 development	0.011-benign	May be involved in maintaining Golgi structure.	[47,48]
*C3*	rs2230199	R102G	c.304C > G	Determinants for COVID-19 prevalence/mortality	0.000-benign	Classical and alternative complement activation pathways are regulated through the activation of C3.	[60,61]
*CCR5*	rs333	S185fs	c.554_585del	Association with susceptibility to SARS-CoV-2 infection and mortality		A beta chemokine receptor family member can cross the membrane seven times. Frame shifts and gene deletions have been associated with HIV infection resistance.	[62]
*IFNAR2*	rs2236757	Intron variant	c.541-50A > G	Significant associations with critical illness		Activated receptor stimulates Janus protein kinases.	[31]
*DPP9*	rs2109069	Intron variant	c.56 + 420C > T	significant associations with critical illness		Gene encodes a protein that is a member of the serine proteases.	[31]
*IFNL4*	rs12979860	Intron variant	g.39738787C > G(T)	It could be a risk factor for the development of COVID-19		Interferons are released in response to viral infection. They block replication and propagation to uninfected cells. IFNL4 encodes the interferon (IFN) lambda 4 protein.	[64]
*GC*	rs59241277, rs113574864, rs182901986, rs60349934, rs113876500	Intron variants	g.34732T > C, g.48235G > T, g.29797C > T, g.49750A > G, g.9899C > T	Polymorphisms are associated with the critical COVID-19 condition		Gene codes transporter for vitamin D that belongs to albumin family.	[65]
*NADSYN1*	rs4944076, rs4944997, rs4944998, rs4944979, rs10898210	Intron variants	g.71211654G > A, g.71206368A > C, g.71207205C > G, g.71197540T > A, g.71210023G > A	Polymorphisms are associated with the critical COVID-19 condition		NAD synthase catalyzes the final step in the biosynthesis of NAD from nicotinic acid adenine dinucleotide.	[65]
*VDR*	rs11574018, rs11574024	Intron variants	g.48297294T > C, g.48296221G > A	Polymorphisms are associated with the critical COVID-19 condition		This gene encodes the vitamin D3 receptor.	[65]
*AGT*	rs699	M259T	c.776T > C	Are associated with the risk of COVID-19 infection, increased risk of infection	1.000-probably damaging	Gene codes angiotensinogen precursor cleaved by the enzyme renin in response to lowered blood pressure.	[46]
*TLR4*	rs4986790	Asp299Gly	c.896A/G	Was associated with severe COVID-19	0.104-benign	Gene codes Toll-like receptor, which plays a fundamental role in pathogen recognition and activation of innate immunity.	[55]
*IL37*	rs3811046	p.Gly31Glu; p.Gly31Ala; p.Gly31Val	c.92G > A; c.92G > C; c.92G > T	Maybe associated with susceptibility to COVID-19 among the Iraqi population	0.484-possibly damaging	The protein is a member of the interleukin 1 cytokine family, and it may be a ligand for the interleukin 18 receptor.	[63]
	rs3811047	p.Thr42Ala; p.Thr42Ser	c.124A > G; c.124A > T		0.000 benign		

**Table 2:** List of mutations engineered into ACE2 protein. Mutations importantly impact interaction with spike protein, as stated in the table. Three mutations on the same molecule—Y27, L330, and L386—increase interaction with the RBD domain of the SARS-CoV-2 spike protein (Uniprot database section: “Mutagenesis”).

Protein ACE2		
Position	Protein Mutation	Function SARS-CoV-2
19	S into P	increases slightly interaction with RBD
24–26	QAK into KAE	slightly inhibits interaction
24	Q into T	slightly increases interaction with RBD
25	A into V	increases slightly interaction with RBD
27	T into Y	increases slightly interaction with RBD
29	L into F	increases slightly the interaction with RBD
31	K into Y	increases slightly the interaction with RBD
34	H into A	increases slightly the interaction with RBD
39	L into R	increases slightly the interaction with RBD
40	F into D	increases slightly the interaction with RBD
41	Y into R	increases slightly the interaction with RBD
42	Q into L	increases slightly the interaction with RBD
69	W into V	increases slightly the interaction with RBD
72	F into Y	increases slightly the interaction with RBD
75	E into K	increases slightly the interaction with RBD
76	Q into T	increases slightly the interaction with RBD
79	L into T	increases slightly the interaction with RBD
89	Q into P	increases slightly the interaction with RBD
90	N into Q	increases slightly the interaction with RBD
91	L into P	increases slightly the interaction with RBD
92	T into Q	increases slightly the interaction with RBD
324	Q into P	increases slightly the interaction with RBD
330	N into Y	increases slightly the interaction with RBD, increases interaction with RBD if associated with Y27 and L386
351	L into F	increases slightly the interaction with RBD
386	A into L	increases slightly the interaction with RBD, increases interaction with RBD if associated with Y27 and L330
389	P into D	increases slightly the interaction with RBD
393	R into K	increases very slightly the interaction with RBD
518	R into G	increases very slightly the interaction with RBD
355	D into A	Restricts interaction with spike [70]

**Table 3:** List of miRNA found in the literature and its function in SARS-CoV-2 disease.

miRNA	Function	Publication
hsa-miR-342-5p	antiviral SARS-CoV2 gene (ORF1ab)	[71]
hsa-miR-432-5p	antiviral SARS-CoV2 gene (ORF1ab)	[71]
hsa-miR-98-5p	antiviral SARS-CoV2 gene (ORF1ab)	[71]
hsa-miR-17-5p	antiviral SARS-CoV2 gene (ORF1ab)	[71]
hsa-miR-17-5p	exhibit experimental evidence of having antiviral roles during infections against SARS1 and SARS2	[72]
hsa-miR-20b-5p	exhibit experimental evidence of having antiviral roles during infections against SARS1 and SARS3	[72]
hsa-miR-323a-5p	exhibit experimental evidence of having antiviral roles during infections against SARS1 and SARS4	[72]
7c-5p	target COVID-19 genome	[29]
miR-27b-3p	target COVID-19 genome	[29]
miR-98-5p	target COVID-19 genome	[29]
miR-125a-5p	target COVID-19 genome	[29]
let-7b-5p	associated with the development of COVID-19 symptoms	[29]
miR-155-5p	associated with the development of COVID-19 symptoms	[29]
miR-186-5p	associated with the development of COVID-19 symptoms	[29]
miR-16-5p	associated with the development of COVID-19 symptoms	[29]
miR-27b-3p	associated with the development of COVID-19 symptoms	[29]
miR-29a-3p	associated with the development of COVID-19 symptoms	[29]
miR-30a-5p	associated with the development of COVID-19 symptoms	[29]
miRs 8066,	associated with host response and virus pathogenicity	[73]
5197	associated with host response and virus pathogenicity	[73]
3611	associated with host response and virus pathogenicity	[73]
3934-3p	associated with host response and virus pathogenicity	[73]
1307-3p	associated with host response and virus pathogenicity	[73]
3691-3p	associated with host response and virus pathogenicity	[73]
1468-5p	associated with host response and virus pathogenicity	[73]
hsa-mir-1267	were found in all five viral SARS-CoV2 cases	[40]
hsa-mir-1-3p	were found in all five viral SARS-CoV2 cases	[40]
hsa-mir-5683	were found in all five viral SARS-CoV2 cases	[40]

**Table 4:** miRNAs binding to SARS-CoV-2 RNA (miRBASE): probability, duplex position, dG, and miRNA-influenced genes are stated.

miRNA	LogitProb	Seed_Position	dG_hybrid	Selected Gene Targets
hsa-miR-3914	0.945766785	4790–4796	−27.6	UBA6, FAM86B2, OSBPL9, GREM1, SLC25A28
hsa-miR-515-5p	0.873907643	1581–1587	−23	ZNF83, PI15, ZNF195, ZNF84, TRAPPC3L
hsa-miR-3934-3p	0.862152158	1403–1409	−32.2	C14orf144, BOK, CDR1as, INHBC, NXF1
hsa-miR-8076	0.820556086	4114–4120	−18.4	CRADD, SLC35E3, RP11-664D7.4, POLR2K, FAM13B
hsa-miR-4502	0.782521425	1875–1880	−20.2	ZNF23, ZNF616, ZNF225, ZNF544, GSG1L
hsa-miR-584-3p	0.759965431	4370–4376	−22.4	IRAK4, IL18RAP, IL22RA2, TIRAP, IL12RB2
hsa-miR-8066	0.758799201	4891–4896	−13.6	IL6ST, IL17A, IL1RL1, IRAK4, IRAK3
hsa-miR-5197-3p	0.751156198	4742–4747	−15.9	IRAK3, IL1A, TIRAP, IL15, IL7R
hsa-miR-1287-5p	0.744896909	1375–1380	−22.9	IL7R, IL10RA, IL27RA, IL1RL1, IL12RB2
hsa-miR-3613-5p	0.731201689	3982–3987	−18	IL25, IL1RL1, IL17A, ILF3, IL1RAPL1
hsa-miR-3611	0.723237424	3015–3021	−18.2	IL17F, IL6ST, TIRAP, IL1RL1, IL26
hsa-miR-148b-3p	0.718940862	2118–2123	−15.8	IL6ST, IL15, IL18BP, SOCS3, DOCK6
hsa-miR-3120-5p	0.709968406	4293–4298	−30.2	IRAK1, IL1A, IL13, IL17A, IL11
hsa-miR-3120-5p	0.703198793	3393–3398	−17.4	IRAK1, IL1A, IL13, IL17A, IL11
hsa-miR-3611	0.696296501	2701–2707	−13.9	IL17F, IL6ST, TIRAP, IL1RL1, IL26
hsa-miR-148b-3p	0.690040719	1299–1304	−25.1	IL6ST, IL15, IL18BP, SOCS3, DOCK6
hsa-miR-3120-5p	0.673755775	3393–3398	−17.5	IRAK1, IL1A, IL13, IL17A, IL11
hsa-miR-148b-3p	0.669885166	902–907	−17.2	IL6ST, IL15, IL18BP, SOCS3, DOCK6
hsa-miR-3120-5p	0.665198433	2291–2296	−19.4	IRAK1, IL1A, IL13, IL17A, IL11
hsa-miR-1287-5p	0.663470144	1176–1181	−22.6	IL7R, IL10RA, IL27RA, IL1RL1, IL12RB2
hsa-miR-584-3p	0.650082156	3307–3312	−17.2	IRAK4, IL18RAP, IL22RA2, TIRAP, IL12RB2
hsa-miR-3691-3p	0.633230969	4612–4617	−15.4	IL1RAP, IL20, IL4R, IL7R, IL5RA
hsa-miR-8066	0.612297577	2022–2027	-13.5	IL6ST, IL17A, IL1RL1, IRAK4, IRAK3
hsa-miR−3613-5p	0.611909431	4978–4983	−15	IL25, IL1RL1, IL17A, ILF3, IL1RAPL1
hsa-miR-3611	0.603752919	2021–2026	−13.4	IL17F, IL6ST, TIRAP, IL1RL1, IL26
hsa-miR-3914	0.601515389	1589–1594	−16	UBA6, FAM86B2, OSBPL9, GREM1, SLC25A28
hsa-miR-8066	0.592544568	3017–3022	−14.7	IL6ST, IL17A, IL1RL1, IRAK4, IRAK3
hsa-miR-5197-3p	0.592355306	4739–4744	−15.7	IRAK3, IL1A, TIRAP, IL15, IL7R
hsa-miR-3120-5p	0.588547281	3257–3262	−23.6	IRAK1, IL1A, IL13, IL17A, IL11
hsa-miR-3611	0.58763097	4698–4703	−19.7	IL17F, IL6ST, TIRAP, IL1RL1, IL26
hsa-miR-515-5p	0.587572941	1482–1487	−19.2	ZNF83, PI15, ZNF195, ZNF84, TRAPPC3L
hsa-miR-3914	0.583849164	1534–1539	−16.7	UBA6, FAM86B2, OSBPL9, GREM1, SLC25A28
hsa-miR-5197-3p	0.580206076	3168–3173	−21.7	IRAK3, IL1A, TIRAP, IL15, IL7R
hsa-miR-148b-3p	0.56875777	714–719	−18.9	IL6ST, IL15, IL18BP, SOCS3, DOCK6
hsa-miR-3914	0.561173741	4006–4011	−13.8	UBA6, FAM86B2, OSBPL9, GREM1, SLC25A28
hsa-miR-378c	0.547705178	1374–1379	−20	CD226, TFCP2L1, PAPOLG
hsa-miR-1287-5p	0.545606444	4056–4061	−14.8	IL7R, IL10RA, IL27RA, IL1RL1, IL12RB2
hsa-miR-148b-3p	0.543191328	115–120	−15.1	IL6ST, IL15, IL18BP, SOCS3, DOCK6
hsa-miR-3611	0.543063193	1984–1989	−13.1	IL17F, IL6ST, TIRAP, IL1RL1, IL26
hsa-miR-8076	0.518454828	1100–1105	−17.8	CRADD, SLC35E3, RP11-664D7.4, POLR2K, FAM13B
hsa-miR-3120-5p	0.503910403	4230–4235	−20.7	IRAK1, IL1A, IL13, IL17A, IL11
hsa-miR-3120-5p	0.502090677	1019–1024	−18.8	IRAK1, IL1A, IL13, IL17A, IL11
hsa-miR-515-5p	0.496816506	357–363	−20.5	ZNF83, PI15, ZNF195, ZNF84, TRAPPC3L
hsa-miR-1468-5p	0.494920764	797–802	−22.4	IL7, IL12B, TIRAP
hsa-miR-1307-3p	0.49446353	676–681	−26.9	IL18, IL20RB, IL10RA, IRAK2, IL6R
hsa-miR-5197-3p	0.441353559	179–184	−22	IRAK3, IL1A, TIRAP, IL15, IL7R
hsa-miR-4502	0.433056019	474–479	−21.5	ZNF23, ZNF616, ZNF225, ZNF544, GSG1L
hsa-miR-3613-5p	0.422107209	2553–2558	−13	IL25, IL1RL1, IL17A, ILF3, IL1RAPL1
hsa-miR-1287-5p	0.395329021	2431–2436	−14.9	IL7R, IL10RA, IL27RA, IL1RL1, IL12RB2
hsa-miR-5197-3p	0.384309463	628–633	−19.4	IRAK3, IL1A, TIRAP, IL15, IL7R
hsa-miR-4502	0.38319915	221–227	−26	ZNF23, ZNF616, ZNF225, ZNF544, GSG1L
hsa-miR-3691-3p	0.37014701	574–579	−20.2	IL1RAP, IL20, IL4R, IL7R, IL5RA
hsa-miR-5197-3p	0.352983944	2484–2489	−14.6	IRAK3, IL1A, TIRAP, IL15, IL7R
hsa-miR-584-3p	0.352800085	495–500	−18.8	IRAK4, IL18RAP, IL22RA2, TIRAP, IL12RB2
hsa-miR-515-5p	0.329015553	267–272	−18.3	ZNF83, PI15, ZNF195, ZNF84, TRAPPC3L

## Data Availability

All the data are included in the paper; any additional data is available from the authors upon request.

## Supplementary Materials

The following supporting information can be downloaded at: https://www.mdpi.com/article/10.3390/biology11020178/s1, Supplementary File S1: Methodology.
