# Supplementary material for: Viral and Host Genetic and Epigenetic Biomarkers Related to SARS-CoV-2 Cell Entry, Infection Rate, and Disease Severity
Source: Biology (Basel). 2022 Jan 23;11(2):178. doi: 10.3390/biology11020178 (PMC8869311; doi:10.3390/biology11020178)
Supplement: Supplementary file 1 [file biology-11-00178-s001.zip › biology-1548539-supplementary.pdf]

Supplementary Materials

Viral and Host Genetic and Epigenetic Biomarkers Related to SARS-CoV-2 Cell Entry, Infection Rate, and Disease Severity

Jernej Gaspersic and Vita Dolzan

Methodology:

Figure preparation

PDB files were downloaded from PDB base: https://www.rcsb.org/ (accessed 15 December 2021). PDB files were opened with a Swiss-Prot PDB viewer, and the 3D structure was presented in ribbons.

Gene ontology analysis

Gene ontology analysis was performed with the WEBGESTALT program, freely available online. Additional settings: Method of interest: Over-representation analysis, Functional database: Gene ontology. We performed three separate tests: Cellular Component, Biological Process, and Molecular function. Reference list: Ilumina humanht 12v3.

miRNA analysis

Into program search miRBASE (mirbase.org), we inserted several times parts of virus RNA (1000 nt) due to size restriction. Settings: Search sequences: mature miRNA, Search method: BlastN, E-value cut off: 10, Maximum no. of hits: 100, Show results only from specific organisms: human has been checked. The program found a new set of 24 miRNAs that bind to virus RNA coding and non-coding regions. The list of miRNA was additionally analyzed with sFold program (https://sfold.wadsworth.org/cgi-bin/index.pl (accessed 15 December 2021)). Settings: Algorithm used: StarMir, Model for prediction: V-CLIP based model (Human), microRNA list was copied, virus RNA sequence pasted. Results of duplex formation and bond energy were presented in the form of tables, figures.
